# A successful management of fungal peritonitis caused by *Rhodotorula glutinis* in CAPD patient, coincident with onychomycosis by *Penicillium* sp: Case report

**DOI:** 10.1097/MD.0000000000034199

**Published:** 2023-07-14

**Authors:** Steven David Panggabean, Ni Made Hustrini

**Affiliations:** a Division of Nephrology and Hypertension, Department of Internal Medicine, Faculty of Medicine, University of Indonesia, Dr. Cipto Mangunkusumo National General Hospital, Jakarta, Indonesia.

**Keywords:** fungal peritonitis, onychomycosis, reinserted catheter, *Rhodotorula glutinis*

## Abstract

*Rhodotorula glutinis* is a rare cause of fungal peritonitis in peritoneal dialysis (PD) patients. The combination treatment between aggressive PD catheter replacement and adequate antifungal treatment is crucial in managing peritonitis due to fungal infection and onychomycosis.

**Patient concerns:**

A 37-year-old man PD patient presented with cloudy effluent, abdominal pain, and black debris in the lumen of his PD catheter. Twelve days before admission, the patient traveled for 10 days to a high-temperature country, Saudi Arabia, for purpose of accomplishing haj. From the physical examinations, there was an onychomycosis in his right toenail.

**Diagnoses:**

The result of the dialysate cell count confirmed the evidence of peritonitis (i.e., cell count of 187 cells per µL, however with polymorphonuclear 31%). The dialysate culture indicated *R glutinis*, with no growth of bacteria. Fungal culture of his toenail scrapings was obtained and the result was *Penicillium* sp.

**Interventions:**

Based on the high clinical suspicion of fungal peritonitis, fluconazole intraperitoneal (IP) was immediately given on the first day in addition to empirical antibiotics, that is, cefazolin and gentamycin IP. His peritoneal catheter was simultaneously removed and reinserted on the 4th day of treatment. Since there was a sign of fluconazole resistance, fluconazole IP was switched into oral voriconazole, without any antimicrobial treatment intraperitoneally. After 21 days of voriconazole, oral itraconazole was given until 3 months for his onychomycosis.

**Outcomes:**

Clinical improvement was seen on the effluent where the leucocyte count falls below 100 cells after 21 days of giving voriconazole.

**Lessons:**

This case report suggests the need for comprehensive evaluations of the risk for fungal infection in continuous ambulatory PD patients, especially those who live in a tropical country.

## 1. Introduction

Fungal peritonitis has a lower prevalence than bacterial peritonitis in patients with peritoneal dialysis (PD),^[1,2]^ however, it has significantly higher mortality.^[2]^ The prevalence of fungal peritonitis in some countries is between 1% and 3% of all-cause peritonitis (<0.01–0.01 episodes per patient year).^[1]^ The highest prevalence of fungal peritonitis had been reported in a tropical country such as India, where the rates were as high as 14.3% of all peritonitis episodes (0.09 episodes per patient year) up to 23.8% of all peritonitis episodes.^[3,4]^ Delayed PD catheter removal was independently associated with mortality in fungal peritonitis patients.^[5]^ Despite technique failure and the mortality rate is still high in early catheter removal,^[6]^ the guideline made a recommendation of immediate PD catheter removal when fungi are identified in PD effluent.^[7]^

*Rhodotorula* is a basidiomycetous yeast in the fungal family *Sporidiobolaceae*; it is an emerging opportunistic pathogen, especially in immunocompromised patients.^[8]^ There were only 11 peritonitis cases related to *Rhodotorula* sp. infections in PD patients that have been reported up to 2017, where most of them underwent catheter removal and transferred to hemodialysis (HD).^[9]^ *Rhodotorula* sp. infections could occur at any site of the body,^[8]^ and *Rhodotorula glutinis* was never reported as an etiology of onychomycosis.^[10]^ We report a case of fungal peritonitis in a PD patient due to *R glutinis*, his Tenckhoff catheter was removed and reinserted simultaneously, and PD could still be maintained. He also had another site of fungal infection in his toenail, which was a possible risk factor for fungal peritonitis.

## 2. Case report

A 37-year-old man presented with cloudy effluent, abdominal pain, and black debris appeared in the lumen of his PD catheter. He has been on continuous ambulatory PD for 5 years after 1 year of undergoing HD as his initial kidney replacement therapy. Chronic glomerulonephritis was identified as the etiology of his end-stage kidney disease (ESKD). No previous PD-related infectious complication was reported and his daily ultrafiltration volume range from 700 to 1200 mL with no urine production. He also presented with hypertension secondary to his kidney disease where blood pressure was well controlled with a combination of calcium channel blocker and angiotensin receptor blocker. He had a previous hepatitis C virus infection which was effectively managed with direct-acting antiviral and confirmed as a sustained virologic response.

**Figure 1.**
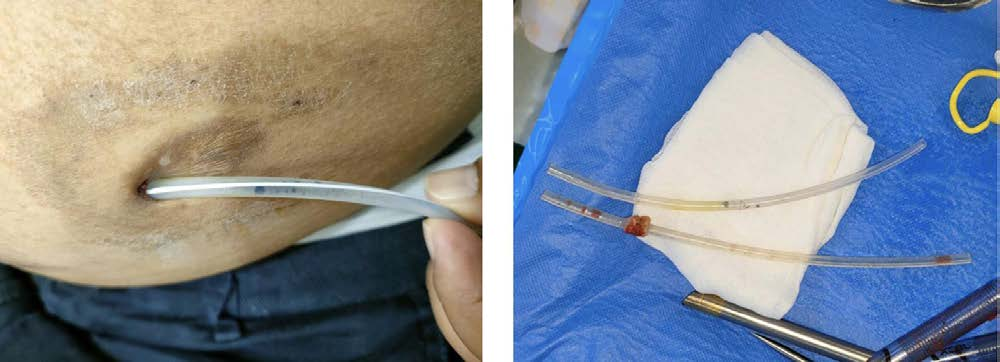
Black debris inside the PD catheter and removal of the catheter. PD = peritoneal dialysis.

Twelve days before admission, the patient traveled for 10 days to a high-temperature country, Saudi Arabia, for purpose of accomplishing haj. On the 5th day of the pilgrimage, black debris appeared inside his PD catheter lumen (Fig. 1), some of them fixed inside the catheter and some others flowed out during draining the dialysate. Two days before the debris appeared he admitted that he washed his hand with water only due to his belief to perform the pilgrimage. Cloudy effluent and abdominal pain appeared later, 2 days before admission. No fever was noticed. No inflow nor outflow obstruction was found, therefore he could maintain good ultrafiltration with no sign of fluid retention.

He had never history of peritonitis before nor recent antibiotic usage. The result of the dialysate cell count confirmed the evidence of peritonitis (i.e., cell count of 187 cells per µL, however with polymorphonuclear 31%). Based on the high clinical suspicion of fungal peritonitis, fluconazole intraperitoneal (IP) was immediately given on the first day of treatment in addition to empirical antibiotics that is cefazolin and gentamycin IP. We simultaneously removed (also seen on Fig. 1) and reinsertion of his peritoneal catheter on the 4th day of treatment. There was no abdominal pain anymore after the surgery. He was then transferred to regular HD for 2 weeks after the new PD catheter reinsertion.

After the catheter removal, we observed no black debris reappeared in his catheter lumen. *R glutinis* organism was confirmed from his dialysate culture, with no growth of bacteria. We continued IP fluconazole as well as an empirical antibiotic for up to 21 days. A series of dialysate cultures and leucocyte counts were performed to monitor the progress of the infection (Table 1).

**Table 1:** Leucocyte count of effluent, culture, and antimicrobial treatment.

	Day-1	Day-4	Day-15	Day-21	Day-25	Day-32	Day-39	Day-44	Day-55	Day-90
Leucocyte count	187 cells/μL		215 cells/μL	279 cells/μL		289 cells/μL	294 cells/μL	124 cells/μL	52 cells/μL	25 cells/μL
PMN	58 cells/μL (31%)		87 cells/μL (40.5%)	213 cells/μL (76.3%)		135 cells/μL (46.7%)	203 cells/μL (69.1%)	22 cells/μL (17.7%)	7 cells/μL (13.4%)	2 cells/μL (8%)
MN	129 cells/μL (69%)		128 cells/μL (59.5%)	66 cells/μL (23.7%)		154 cells/μL (53.3%)	91 cells/μL (30.9%)	102 cells/μL (82.3%)	45 cells/μL (86.6%)	23 cells/μL (92%)
Culture of effluent	*Rhodotorula glutinis* No bacteria growth				Sterile			Sterile		
Culture of Tenckhoff catheter		Sterile								
Antibiotic	Cefazolin 1 × 1 g + Gentamicin 1 × 40 mg, intraperitoneal									
Antifungal	Fluconazole 1 × 200 mg, intraperitoneal				Voriconazole 2 × 200 mg, per oral					

MN = mononuclear, PMN = polymorphonuclear.

**Figure 2.**
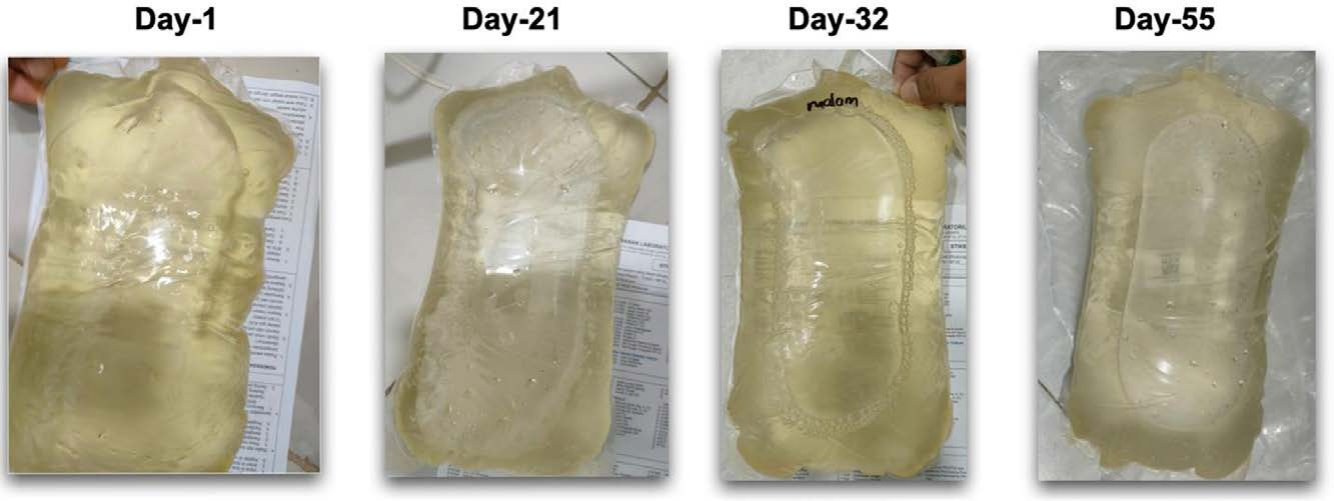
Progression of the effluent.

We observed that there was no growth of any microorganisms after the catheter removal; however, we found no significant improvement in the clarity (Fig. 2) and leucocyte count of the effluent 3 weeks after adequate antimicrobial treatment despite the relatively stable clinical condition.

We considered that this condition might be related to a fluconazole-resistant status. Therefore we switched fluconazole IP to oral voriconazole, without any antibiotic treatment. Oral voriconazole 200 mg twice daily was started and administered for 21 days. We observed a significant clinical improvement in the effluent where the leucocyte count fall below 100 cells per µL.

**Figure 3.**
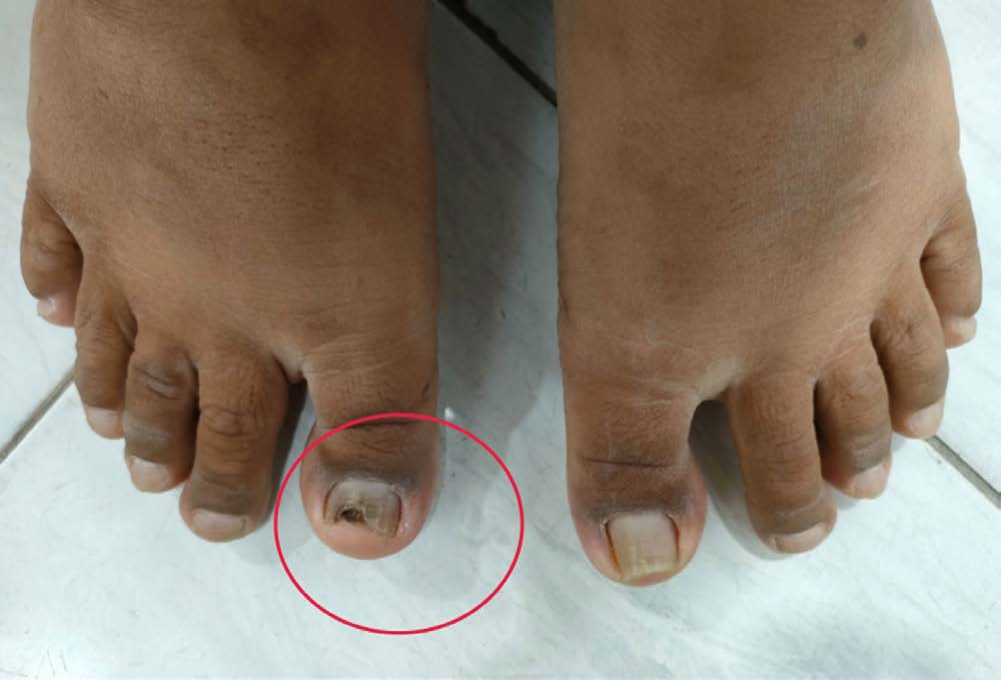
Discoloration and nail plate destruction.

**Figure 4.**
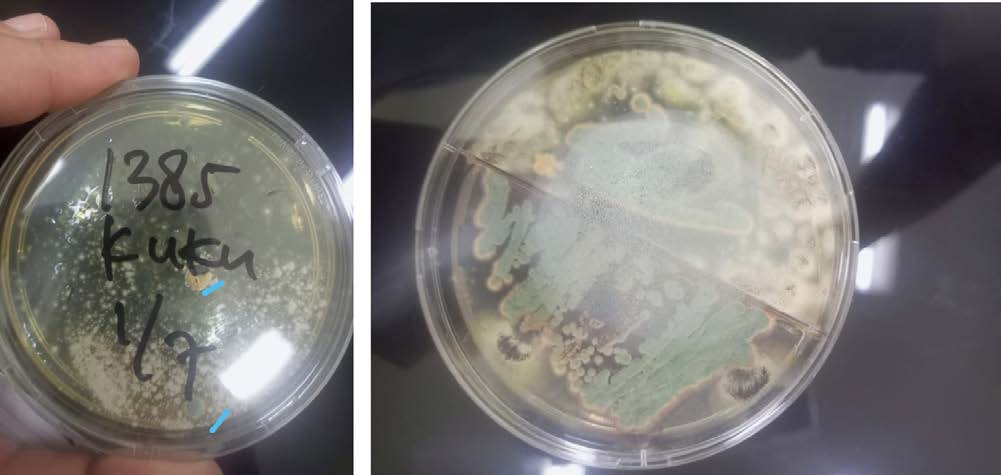
*Penicillium* sp. from fungal culture of toenail scrapings.

We explored the possible risk factors for fungal peritonitis in this patient and we found that he had onychomycosis on his right toenail. There was discoloration and nail plate destruction on his right toenail (Fig. 3). The patient admitted that he unintentionally touched his toenail when performing the dialysate exchange for some time. We did a fungal culture of his toenail scrapings and demonstrated *Penicillium* sp. microorganism (Fig. 4).

Oral itraconazole was the sensitive antifungal treatment based on his nail’s fungal culture, and was administered for approximately 3 months.

## 3. Discussion

The highest prevalence of fungal peritonitis was reported in India (14.3%–23.8% of all peritonitis episodes).^[3,4]^ The contribution of fungal peritonitis on PD technique failure and mortality remains high.^[6]^ There is a wide spectrum of fungi as causative organisms of fungal peritonitis, that is yeast, hyaline mold, and non-hyaline mold, with approximately 80 fungal species being potential causative organisms.^[11]^ Candida belongs to the yeast family and is the predominant cause (65.1%–100%) of fungal peritonitis.^[2–4,6,12]^ Our case demonstrated a *R glutinis*-related PD peritonitis that was successfully managed with voriconazole and catheter removal.

*Rhodotorula* is a basidiomycetous yeast in the fungal family *Sporidiobolaceae*. It is widespread and commonly found in air, ocean water, lakes, soil, fruit juice, and milk.^[8,13]^ *Rhodotorula* is a temporary commensal microorganism of the nails, skin, gastrointestinal, urinary, and respiratory tracts in humans.^[13,14]^ In the past this yeast was considered a nonpathogenic microorganism, but now it has emerged as an opportunistic causative agent, especially in immunocompromised patients.^[14,15]^

There were various clinical presentations of *Rhodotorula* infections such as central venous catheter-associated infections, fungemia, endocarditis, ocular infection, central nervous system infections, and peritonitis in PD patients. Most of the patients (87%) with *Rhodotorula* infection had underlying immunosuppressive conditions (i.e., prolonged corticosteroid use, acquired immunodeficiency syndrome, neutropenia, and malnutrition) and cancer.^[8]^

There were 11 cases of peritonitis PD patients caused by *Rhodotorula*, 4 of them (36.3 %) had specific immunocompromised conditions (2 patients with diabetes mellitus, 1 patient with immunosuppressive therapy, 1 patient with human immunodeficiency virus infection).^[9]^ Our patient had no history of diabetes mellitus nor immunosuppressive therapy, his human immunodeficiency virus screening was negative, and he also had no leucopenia or neutropenia. But it is important that we have to consider that ESKD patients are immunocompromised patients.

In ESKD patients, both defective renal metabolic activities and impaired glomerular filtration result in the accumulation of uremic toxins interfering with the immune system. The proneness of ESKD patients to have infections due to the decreased phagocytosis of polymorphonuclear leucocytes (eosinophil, basophil, and neutrophil) is caused by factors such as uremic toxins, iron overload, and anemia of renal disease. Neutrophils have an essential role in the defense against bacterial and fungal infections.^[16]^

The genus *Rhodotorula* includes 8 species, some of which can cause disease in humans such as *Rhodotorula mucilaginosa, R glutinis, Rhodotorula rubra*, and *Rhodotorula minuta*. Two species that is *R glutinis* and *R minuta* are less frequently isolated from natural environments, compared to *R mucilaginosa*.^[13]^ Two species of *Rhodotorula* had been reported in 11 peritonitis PD patients, *R mucilaginosa* in 4 patients, and *R rubra* in 7 patients.^[9]^ Our PD patient demonstrated *R. glutinis* that caused peritonitis, the first ever reported case of peritonitis PD patient. Although it was rare, *R. glutinis* had been reported in other site infections such as fungemia, ocular infection, and central nervous infection cases.^[8]^

There are some potential causes of black-stained PD tubing except for peritonitis. Contamination of spilled-out povidone-iodine solution during transfer set change might be the frequent cause. Other potential causes were inadequate dialysis, low serum albumin, transfer set soaking with antiseptics, dressing technique, and the existence of dry abdomen period.^[17]^

Removal and reinsertion of the PD catheter were done simultaneously based on consideration of clinically stable condition, adequate PD, and no sign of systemic infection. A study of 11 cases of *Rhodotorula* infection in peritonitis PD patients found that the majority of patients conducted catheter removal (7 patients), 2 patients were simultaneously removed and reinserted the catheter, and 2 patients had no catheter removal. One of the patients that received a simultaneous procedure continued successful PD like our patient, but another patient had bowel adhesion and dialysis failed furthermore.^[9]^ In our opinion for a peritonitis PD patient caused by *Rhodotorula* infection, it may be considered to do simultaneous catheter removal and reinsertion if the clinical patient condition is stable.

International Society of Peritoneal Dialysis guidelines recommend voriconazole as an alternative antifungal treatment for fluconazole-resistant species,^[7]^ but until now there is no report concerning voriconazole treatment associated with *Rhodotorula* sp. infection both in PD-related infections or at any site of infections.^[8,9]^ We chose to administer voriconazole as the alternative treatment after an assumption of a fluconazole-resistant state. Oral voriconazole penetrates well into peritoneal fluid with minimal peritoneal clearance, therefore no dosage adjustment is needed for PD patients.^[18]^

Eight patients out of 11 cases reported with *Rhodotorula* peritonitis in PD patients were treated with Amphotericin B.^[9]^ We did not use Amphotericin B due to its nephrotoxic effect and intravenous administration, while our patient was stable therefore no need for the hospital stay.

Our case is the first report of a successful *Rhodotorula*-associated PD peritonitis treated with voriconazole. Fungal peritonitis has a high mortality rate and technical failure, even though there are improvements after catheter removal.^[5,6]^ In serial 11 cases of *Rhodotorula* peritonitis, only 1 died by aspiration pneumonia, not because of peritonitis complication.^[9]^ In systemic infection of *Rhodotorula*, the mortality rate of fungemia was as high as 14.4%.^[8]^ We found that in our patient after the catheter removal, his condition was clinically stable, although the effluent cell count was improved slowly. Catheter removal remains the key point in the management of *Rhodotorula* peritonitis in PD patients to prevent mortality.

Our patient did not have common predisposition factors for fungal peritonitis in PD patients. His onychomycosis was another site of fungal infections, but we could not conclude it was the risk factor because the fungal culture of toenail scrapings of our patient demonstrated *Penicillium* sp., different from his effluent. We planned oral itraconazole 200 mg per day for 3 months based on the antifungal resistance test. *R. glutinis* infection of the nail was reported in a 74-year-old woman. Oral itraconazole was given for her antifungal therapy and clinical recovery was detected.^[10]^

There are no studies that reported the correlation between other sites of fungal infection such as onychomycosis with the incidence of fungal peritonitis in PD patients. Data from HD patients, the prevalence of onychomycosis was higher than in the general population.^[19]^ Toenail scraping cultures of fungal were positive in 19.7% of patients with dystrophic nail changes in HD patients.^[20]^ Considering the immune dysfunction due to uremia in HD and PD patients, the study of onychomycosis in PD patients is essential because it may be one of the risk factors of fungal peritonitis.

## 4. Conclusion

This is the first report of *R glutinis*-associated PD peritonitis successfully managed with an aggressive PD catheter replacement and adequate antifungal treatment. Comprehensive evaluations of the risk for fungal infection in continuous ambulatory PD patients are necessary especially those who live in a tropical country.

## Author contributions

**Conceptualization:** Steven David Panggabean, Ni Made Hustrini.

**Data curation:** Steven David Panggabean.

**Investigation:** Steven David Panggabean, Ni Made Hustrini.

**Methodology:** Ni Made Hustrini.

**Validation:** Steven David Panggabean, Ni Made Hustrini.

**Visualization:** Steven David Panggabean.

**Writing – original draft:** Steven David Panggabean.

**Writing – review & editing:** Ni Made Hustrini.
